# Testing the somatic marker hypothesis in decisions-from-experience with non-stationary outcome probabilities

**DOI:** 10.3389/fpsyg.2023.1195009

**Published:** 2023-07-28

**Authors:** Rebecca J. Wright, Tim Rakow

**Affiliations:** Department of Psychology, University of Essex, Colchester, United Kingdom

**Keywords:** risky choice, experiential choice, learning, reversal learning, skin conductance, outcome response, anticipatory response

## Abstract

**Introduction:**

The Somatic Marker Hypothesis (SMH) posits that in experience-based choice, people develop physiological reactions that mark options as either positive or negative. These somatic markers aid decision making because they differentiate between “good” and “bad” options during pre-choice deliberation.

**Methods:**

We examined this proposed role for somatic states in two decision-from-experience tasks (each *N* = 36) in which participants selected repeatedly with full feedback (i.e., for obtained and forgone outcomes) between two unlabeled options that returned wins or losses, with half receiving an additional summary of past outcomes. The probabilities of good and bad outcomes changed at an unannounced point. Participants completed a 100-trial game with a switch in the optimal option after trial 40 (Study 1) or a 200-trial game with switch points after trial 40 and trial 120 (Study 2). Skin conductance (SC) was measured continuously as an index of emotional intensity, from which we extracted measures of anticipatory SC (pre-choice) and outcome SC (post-choice).

**Results:**

Participants reliably selected the optimal option prior to any switches. They also altered their choices appropriately when the payoffs changed, though optimal play following payoff switches was reduced. Losses resulted in a greater outcome SC than wins, but only in Study 1, as did the finding that the outcome SC was greater when the forgone outcome was positive. Anticipatory SC did not reliably predict optimal play in either study.

**Discussion:**

These results provide little support for the SMH. Our studies point to the importance of using diverse tasks and measures and very large sample sizes when testing the role of somatic states in decision making.

## Introduction

In decisions-from-experience, one learns about the options by observing past outcomes (Rakow and Newell, 2010). The research tasks used to investigate how people make such decisions reflect a variety of everyday decisions. For example, Lejarraga et al. (2016) showed participants the day-by-day outcomes for different treatment options that differed in their (observed) rate of side effects. Lejarraga et al. (2016) compared participants' treatment preferences with choices for structurally equivalent decisions involving monetary outcomes. Liang et al. (2022) used a microworld to examine decisions in response to rare disasters, comparing reactions to disasters experienced first-hand with disasters observed at a distance (either near or far). In the context of interpersonal decisions, Denrell (2005) noted that the impressions we form about others often arise from samples of observed interactions, which then guide our decisions about who to interact with.

One feature of real-world decisions-from-experience is that the world is not static: the climate changes, financial markets boom or bust, and people or organizations change. Thus, while past events can be a guide to what to expect in the future, the world may change such that successful courses of action become suboptimal. In this study, we use a laboratory task to examine whether and how experience-based choices adapt to such changes, with a specific focus on the role of somatic states in such choices. In doing so, we build on previous investigations that have used the Iowa gambling task to examine the somatic marker hypothesis.

### Reversal learning in the Iowa gambling task and other decision-from-experience tasks

The Iowa gambling task (IGT; Bechara et al., 1994) is an example of decision-making from experience. In the IGT, wins and losses from card draws from four decks must be *experienced* to work out which decks are advantageous. The IGT was devised to test the somatic marker hypothesis (SMH), which posits that emotions play a key role in decision-making under uncertainty (Damasio, 1994). Specifically, as a result of their previous choices, people develop physiological reactions that mark options as either positive or negative. These somatic markers then differentiate “good” options from “bad” ones when those options are considered again. In time, this mechanism guides people away from making bad choices.

The original version of the IGT was set up so that the large losses in a disadvantageous deck with infrequent losses occur relatively late in the deck, thereby giving the initial impression of it being an advantageous deck. To play optimally, participants must therefore perform reversal learning—changing their behavior when an ostensibly advantageous deck is seen to be disadvantageous. Poor reversal learning has been found in patients with impairment to the ventromedial prefrontal cortex (VMPFC). This could, therefore, explain their poor performance on the IGT (Dunn et al., 2006).

Successful (reversal) learning in decisions-from-experience requires response inhibition. For example, to succeed at the IGT, one must inhibit a win–stay/lose–shift response pattern (Restle, 1958) when experiencing a loss from an advantageous deck. VMPFC patients' failure to pick from the advantageous decks could reflect the inability to inhibit this response. To investigate this, Fellows and Farah (2005) shuffled the card order of the IGT decks so that the disadvantageous decks were no longer initially the better decks. Fellows and Farah found that the task performance of VMPFC patients became similar to that of control participants. They concluded that poor IGT performance in this patient group was primarily due to impaired reversal learning. Consistent with this, after examining participants' conscious knowledge in the IGT, Maia and McClelland (2004) concluded that—contrary to the SMH—there was no need to use non-conscious somatic markers to guide choice. Therefore, the poor IGT performance of VMPFC patients could be better explained by poor reversal learning.

However, in response to Maia and McClelland (2004), Bechara et al. (2005) argued that reversal learning is not the only requirement for successful performance in the IGT. Rather, they proposed that the SMH could explain reversal learning via the development of a somatic “stop signal” in response to experiencing bad outcomes from card draws. These markers are greater before selecting from the disadvantageous decks, due to the poorer outcomes experienced, and this facilitates the shift to picking more from the advantageous decks. Further support that reversal learning is not the only necessary skill for successful IGT performance comes from Turnbull et al. (2006). People with schizophrenia have been shown to have difficulty with flexible behaviors, but performance in their emotion-based learning has more mixed results. To assess both these types of abilities in this patient group, Turnbull et al. (2006) employed the IGT. Participants initially played 100 trials of the original IGT but the good decks then shifted in three further 40-trial phases: Decks A and D, A and B, and B and C successively became good decks during the three shift periods. The participants with schizophrenia played advantageously (comparable to controls) in the first 100 IGT trials. However, performance during the shift phases was near chance-level for the patient group with the most severe negative symptoms, thereby suggesting that good performance on the IGT can be acquired in the presence of poor reversal learning.

In this study, we use a two-option decision-from-experience task where the probability distribution is *non-stationary*. Thus, the probabilities of good/poor outcomes change at pre-determined (but unsignalled) points during the task. Additionally, we measure skin conductance responses (SCRs) to examine the role of somatic states in a task that requires reversal learning. Because it is debated how much reversal learning is needed in the IGT (Maia and McClelland, 2004, 2005; Bechara et al., 2005), utilizing a simpler decision task with a defined point at which the optimum option changes should help to identify the utility of somatic states in reversal learning. In doing so, our investigation adds to the relatively small body of research that uses a decision task other than the IGT to test the SMH (e.g., Wright and Rakow, 2017). This is important because any theory of everyday decision-making under uncertainty should undergo tests using a range of tasks so that the generalizability and boundary conditions of the theory are established. Another key feature of our investigation is that we measure *anticipatory* SCRs to test predictions from the SMH. Given the SMH's proposed role for somatic states in *guiding choice prospectively* and the importance of the SCR data that—almost 30 years ago—brought the SMH to prominence (e.g., Bechara et al., 1994), it reflects poorly on the field that such data are relatively sparse. For example, a meta-analysis by Simonovic et al. (2019) identified only 20 IGT studies in non-clinical populations (published in 16 articles) that used anticipatory SCRs to test the SMH (this, from 3,999 articles that mentioned the IGT in the title or abstract). Therefore, our paper is valuable because it adds two further studies using anticipatory SCRs to this small but important body of SMH research. A further key feature of our investigation is that we manipulate (between-subjects) the presence of some descriptive information about the option outcomes. This allows us to examine whether and how descriptive information moderates preference and/or the role of somatic states in decisions-from-experience.

### How decision processes differ between decisions-from-experience and decisions-from-description

After considering a description of two options, Option A offering a 90% chance of nothing and a 10% of £10 and Option B offering £1 for certain, most individuals choose gamble A. This choice reflects a general pattern: when faced with *described* gambles having small probabilities for the most extreme outcome, most individuals are risk-seeking for gains and risk-averse for losses. Prospect theory (Kahneman and Tversky, 1979) explains such preferences via *as-if* decision weights, which are transformations of the probabilities for each outcome. Each decision weight is multiplied by its respective outcomes to compute an overall value for each outcome. According to prospect theory, small probabilities are *as-if* overweighted. This overweighting makes gambles with a small probability of a large gain highly attractive and makes gambles with a small probability of a large loss very unattractive.

However, this pattern of preference often reverses if the option payoff distributions are *experienced* as a sequence of observations—such as by drawing cards in the IGT or by selecting from on-screen buttons or “money machines” to reveal outcomes. In such decisions-from-experience, most individuals prefer Option B in the above example. Barron and Erev (2003) highlighted this “description–experience gap,” which implies the *underweighting of rare events* in decisions-from-experience.^1^ This description–experience gap has since been well-documented (Rakow and Newell, 2010; Wulff et al., 2018) including for some choices that do not involve small probabilities (Ludvig and Spetch, 2011). However, the reasons for this description–experience gap remain a point of debate (Wulff et al., 2018). One possibility is that the neural or somatic representation of outcomes and/or options differs between decisions-from-description and decisions-from-experience (Glöckner et al., 2012). Thus, our tests of the distinctive pattern of somatic responses that the SMH predicts for decisions-from-experience have implications for a wider debate about the processes at work in decisions-from-experience.

### Decisions-from-experience with non-stationary payoffs

The majority of studies examining repeated decisions-from-experience utilize gambles for which the probabilities of the outcomes remain stable across all trials (i.e., *static probabilities*). However, some studies have employed non-stationary distributions, where the probabilities of the payoffs change within the task. These non-stationary payoff distributions are akin to what happens in the original IGT, where Deck B initially appears advantageous because no loss is experienced on the first few deck selections.

Biele et al. (2009) examined dynamic decision environments using one-armed restless bandit problems. Participants chose between a stable safe prospect with a constant medium payoff of 0 and an unstable risky prospect with a payoff of either +1 or −1 depending on its state. The state was determined using a two-state Markov process. If the state was positive at trial t, it remained positive at t + 1 with probability p (either 0.95/0.50 in Study 1); and if the state was negative at trial t, it remained negative at t +1 with probability q (0.05/0.50). Participants were highly sensitive to the changing probabilities of the payoffs, achieving near-optimal performance. Similar to decisions-from-experience in static environments, underweighting of small probabilities and payoff variability was also observed, as inferred from models fitted to the data, suggesting a reliance on small samples of experiences.

Rakow and Miler (2009) examined choices in repeated-choice games with non-stationary payoff distributions. Their participants chose between two “money machines” for either 60 or 100 trials, to obtain as many points as possible. The possible win and loss amounts were stated at the start of the game, and participants saw both the obtained and forgone outcomes of each money machine. One game included a stationary option with a 70% of winning 10 points, otherwise a 30% chance of losing 20 points. The non-stationary option started with a 90% of winning 10 points (otherwise losing 20 points), which reduced to 50% at trial 20 onwards. Participants were not informed of the options' probability distributions, although they were informed that the options could *change* over the course of the game. In each game, one machine's probability changed gradually at a rate of either 0.01 per trial over 40 trials (Study 1) or 0.02 per trial over 20 trials (Study 2). It varied between games when this change began (trial 20 vs. 40) and its direction (increasing vs. decreasing win-probability). In one condition, participants also had a running history for past outcomes; participants were shown cumulative totals for the number of times each machine had delivered a win or loss amount. The results demonstrated reasonably rapid initial learning whereby, after the first few trials, participants generally selected the machine with the better payoffs, but with a slower adaption to pick from the better option after a switch in the payoffs. Providing participants with a history improved the initial learning to pick the optimal machine, but it sometimes hindered the adaption to the subsequent changes in the payoffs compared with when no history was provided.

Our two studies, reported here, follow a similar design to examine the role of somatic markers when, initially, a non-stationary option stochastically dominates a stationary option but changes to be suboptimal relatively early in the game. This mimics what happens with Deck B in the IGT. Study 1 uses a 100-trial task with a switch in the optimal option at trial 40, and Study 2 uses a 200-trial task with switch points at trials 40 and 120. The processes posited by the SMH predict successful initial learning *and* subsequent reversal learning in such tasks, and this is tested in our two studies.

According to the SMH, people develop “hunches” about the options they experience, and via somatic responses (which we index via SCRs) mark these options as either positive/safe or negative/risky. This promotes successful decision-making by gradually guiding decision-makers away from repeating bad decisions. In our two studies, we examine whether participants develop a preference for the (current) optimal option and whether this is aided by developing greater anticipatory markers for the suboptimal option. We are also interested in whether participants adapt to a change in an option's payoff distribution, and whether this is also reflected in the anticipatory, and outcome, skin conductance (SC). The SMH predicts that somatic markers develop and are greater for the inferior option. Therefore, after a change in the payoff structure, the SMH predicts that somatic markers will develop and be greater for the new, currently worse, option.

The task that we use presents participants with both the outcome obtained from each choice and the forgone outcome for each trial's unselected option. This allows our participants to experience regret because they can see what they would have obtained from a different course of action. This is a valuable feature of our study because experienced regret has been theorized (Loomes and Sugden, 1982) and found to be an important driver of various kinds of decisions (Zeelenberg and Beattie, 1997; Zeelenberg and Pieters, 1999, 2004; Kareev et al., 2014). Zeelenberg (1999) highlighted that the adaptive function of experienced regret may be to exacerbate the misfortune felt from our mistakes, to help us learn from them. This parallels the assumptions of the SMH that emotional signals help to bias us away from repeating previous poor decisions.

## Study 1

Study 1 employs a 100-trial non-stationary decisions-from-experience task adapted from Rakow and Miler (2009) with an immediate switch point at trial 40, where the non-stationary option changes from optimal to suboptimal choice. We also provide a running history of past outcomes to half of the participants.

Based on Rakow and Miler (2009), we expect that participants will have developed a clear preference for the optimal non-stationary option within the first 40 trials but will adapt only partially to the change in payoffs by the end of the task:

**H**_**1**_**:** There will be a preference for the optimal option by the second block of 20 trials.

**H**_**2**_**:** The number of selections from the optimal option for trials 81–100 will be less for trials 21–40.

Rakow and Miler (2009) also found that providing a running history of each option's outcomes sometimes hindered participants' adaption to a change in which option was optimal. We therefore test whether:

**H**_**3**_**:** Participants provided with a history of the options' outcomes adapt more slowly following the switch, and have fewer selections of the optimal option in the last 60 trials than participants without the history.

Hypothesis H_1_ supports the somatic marker hypothesis (SMH), which also predicts reversal learning (though it is unclear to us whether, as we predict in H_2_, this will be slower than the initial learning). Because we collect skin conductance (SC) data, we can also test the SMH's explanation for *how* learning occurs. The SMH posits that in response to positive and negative outcomes experienced after selecting options, emotional biasing signals mark options (Damasio, 1994). Participants should therefore have *outcome* SCRs that are greater for losses (“punishments”) than for wins (“rewards”). Therefore, based on the SMH, we predict:

**H**_**4**_**:** Outcome SC will be greater for negative outcomes (−10) than for positive outcomes (+10).

As participants will also see the forgone outcome (for the option they did not pick), we will also examine whether the outcome of the chosen option *relative to* the forgone option's outcome affects the outcome SC. Obtaining a negative outcome (−10) when the forgone option was positive (+10) should result in a higher outcome SC, reflecting an elevated emotional response reflecting the regret of a missed reward (e.g., Astor et al., 2011):

**H**_**5**_**:** Obtaining a negative outcome when the forgone outcome is positive will result in greater outcome SC compared with other combinations of obtained and forgone outcomes.

The SMH's key prediction is that over the course of the task, the outcome responses aid the development of *anticipatory* SCRs that mark options. The SMH predicts greater anticipatory SC before selecting disadvantageous options than before selecting advantageous options. We examine anticipatory SC and its role in optimal selections in the two phases of the task—before and after the switch. We, therefore, test this prediction derived from the SMH:

**H**_**6**_**:** Anticipatory SC responses develop and are greater for selections of the suboptimal option (whichever option that is for a given phase of the task).

### Method

#### Participants

Participants were recruited from the University of Essex, Psychology Department's Volunteer list,^2^ which included university students (the majority) or staff. There were 36 participants (23 female) with a mean age of 24.33 years (*SD* = 3.80, range 19–35, IQR 22–26). Two participants were excluded due to either not following instructions correctly or apparatus error and were replaced. This sample size is typical for studies that test the SMH via SC data; a meta-analysis by Simonovic et al. (2019) reports that IGT studies with anticipatory SC data for non-clinical populations had a median *N* = 40.5 (IQR 32–70).^3^

#### Apparatus

The SC activity was recorded using a Mind Media NeXus-10, a multi-channel physiological monitoring and feedback platform, with a sampling rate of 32 samples per second. SC activity was recorded continuously throughout the study and for critical events in each task, a trigger was sent via a button box to mark the SC reading. The SC data were then analyzed using Ledalab, MATLAB-based analysis software designed for SC data analysis (Benedek and Kaernbach, 2010). Continuous decomposition analysis was used, with no downsampling of the data. All data were optimized (Ledalab optimizes data for each participant individually). The minimum amplitude threshold was set to 0.01 muS. Data were exported using SCR event-related activity, 1–4 s after an event for the outcome SC and from 2 s before up until a trigger for anticipatory SC.

#### Materials and design

A computerized decisions-from-experience task was created in Real Studio, which sent event triggers compatible with the NeXus-10 during the task. On each trial of the “game,” participants selected between two “money machines” represented as on-screen buttons. Both machines paid out a win of +10 or a loss of −10 but with different probabilities of a win. One machine (stationary option) had a probability of 0.5 of winning, fixed for all 100 trials. The other machine (non-stationary) had a probability of 0.7 of winning for the first 40 trials, which switched to a 0.3 probability at trial 41, at which it remained for all subsequent trials. Participants completed the task after two other risk-taking tasks (not reported here). Participants were randomly assigned to either the experience-only or experience-plus-history condition.

#### Procedure

On arrival at the laboratory, participants read a consent form explaining the payment for their participation and details of the NeXus-10 equipment. Once consent was obtained, using the participants' non-dominant hand, the electrodes were attached to the distal phalange of the first and third digits. To do so, participants cleaned the palm side of their first and third fingertips with an alcohol wipe and the experimenter applied opaque adhesive paste. The sensors were attached, and then, the participant placed their hand palm upwards on a cushion on the desk aiming to keep it as still as possible throughout the experiment. As recommended, the sensors were given 5 min to settle (Figner and Murphy, 2011). A measure of baseline activity was taken to control for individual differences in SC before the start of a task. This was calculated as a ratio of 1:5 of the average time taken to complete the task, which resulted in 3 min of baseline activity recorded.

Before commencing the task, participants were given standardized instructions (see Appendix) which outlined that points could be won or lost on each “go” of the “game” and that win/loss probability could change. The inter-trial interval of the task was set to 6 s to allow for suitable SC recording. Participants used the computer-mouse to select machines. Both machines displayed the outcome (win or lose) on every trial; the machine selected would display “You have won/lost 10 points.” The unselected machine would display “You would have won/lost 10 points.” In the experience-plus-history condition, the win and loss history for each machine was displayed above each machine. This history displayed the previous number of times the machine had won and lost and updated every trial. Once participants had played all 100 trials, their total points remained on screen. The study session lasted ~50 min, for which participants received UK£5 (or course credit).

#### Measures and data analysis techniques

The measure of SC reported here, and in Study 2, was the mean phasic driver within the response window. Ledalab documentation states that this variable “represents phasic activity within the response window most accurately, but does not fall back on classic SCR amplitudes” (http://www.ledalab.de/documentation.htm, see also Benedek and Kaernbach, 2010). The recording interval from 1 to 4 s after a trial outcome appeared was used for outcome SC, and the 2-s interval before selecting the option was used for the anticipatory SC. Due to the repeated-measures design, all regressions were run using a multilevel random intercepts regression model (Nezlek et al., 2006). Multilevel models are used to assess data that contain a natural hierarchy or clustering of cases within variables. This is appropriate with the current data because the 100 selections (200 in Study 2) represent a cluster of observations for each participant. Multilevel models differ from standard regression models (e.g., ordinary least squares) due to dividing the error variance into separate components. This allows the model to control for the patterns of the structured data: patterns in the error from the model are assumed to have a reliable structure and are not just noise. This technique allows the examination of trial-by-trial data in a principled fashion (e.g., by not treating trials as independent observations).

Research has shown both inter- and intra-individual variability in the rise and recovery time of SCRs (Edelberg and Muller, 1981; Breault and Ducharme, 1993). The anticipated variation between participants in SC (Figner and Murphy, 2011) was accounted for by entering participants as a level 2 random intercept within the multilevel model. Multilevel modeling was utilized to distinguish within- and between-participant variations in SC (Goldstein, 1995; Hox, 2010). The level 1 variables were at the individual trial level (100 data points in Study 1 and 200 in Study 2) and included participants' selections (e.g., which option they picked and whether the selection was optimal) and SC measures (outcome SC; anticipatory SC). We checked for skew in continuous dependent variables for each participant individually. Outcome SC was found to be positively skewed, so was log_10_ transformed for all participants, in both Study 1 and Study 2. Any marginal means reported for outcome SC are log_10_-transformed marginal means. We checked for outliers in all regressions using the Blocked Adaptive Computationally efficient Outlier Nominator (BACON; Billor et al., 2000) procedure, which identifies multivariate outliers in a set of predictor variables and removed those outliers from all regression analyses.

### Results

For data presentation and analysis, we grouped trials into five 20-trial blocks. To examine whether participants were picking more from the optimal (non-stationary) option by the second block (21–40 trials), a one-sample *t*-test was conducted which found that the mean number of selections from the optimal (non-stationary) option in block 2 (*M* = 13.78) was significantly >10 (chance-level performance), *t*_(35)_ = 4.44, *p* < 0.001, *d* = 0.74. This fairly large effect supports H_1_ that participants would develop a preference for the optimal option within the first 40 trials. A paired-samples *t*-test comparing the number of optimal selections (from the stationary option) in block 5 (*M* = 10.36) to the optimal selections (from the non-stationary option) in block 2 (*M* = 13.78) revealed—in support of H_2_–significantly fewer optimal selections in block 5 than in block 2, *t*_(35)_ = 2.56, *p* = 0.015, *d* = 0.43.

**Figure 1:**
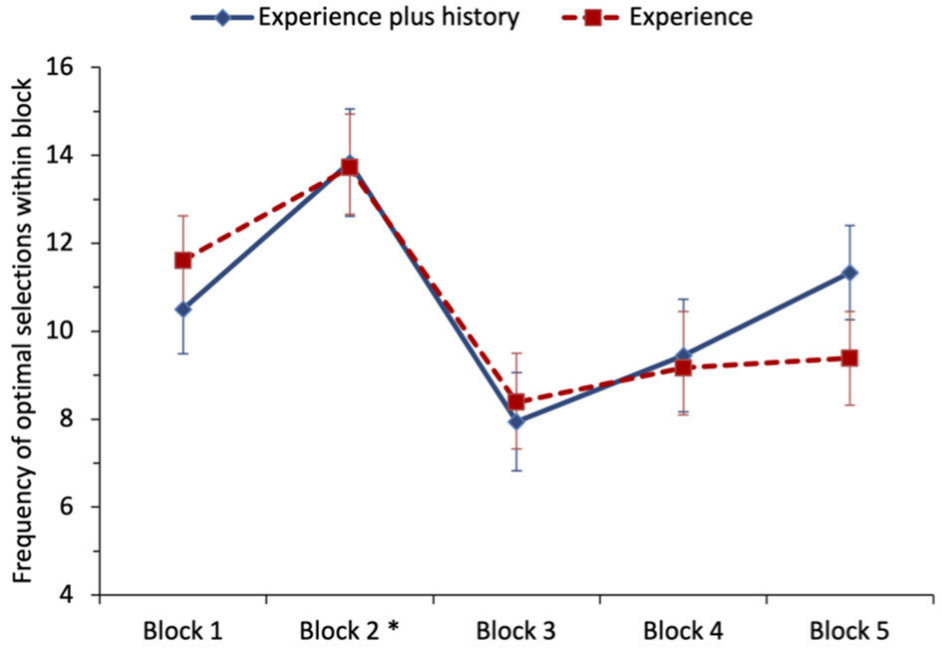
Study 1: mean frequency of selection of the optimal option (non-stationary option in blocks 1 and 2, stationary option in blocks 3–5) for each condition of the task. The error bars show the standard error of the mean. The asterisk denotes that a switch in payoffs occurred at the end of block 2.

To examine the effect of having the running history in addition to experiencing the outcomes, the number of times the optimal option was selected was analyzed as five blocks of 20 trials (coding specific to whichever option was optimal in a given block). A repeated-measures ANOVA was performed with the number of times the optimal option was picked as the dependent variable and block and condition (experience-plus-history vs. experience-only) entered as factors, see Figure 1. Mauchly's test for sphericity was statistically significant for block; we report Greenhouse–Geisser corrected degrees of freedom for all ANOVA effects for block (also in Study 2). The main effect of block was medium-to-large and statistically significant, *F*_(1.75, 59.55)_ = 6.73, *p* = 0.003, $\eta_{p}^{2}$ = 0.165. Tukey's *post-hoc* tests revealed a statistically significant increase in optimal selections from block 1 (*M* = 11.06) to block 2 (*M* = 13.78). There was a statistically significant decrease in optimal selections from block 2 (*M* =13.78) to block 3 (*M* = 8.17), the first block of trials after the switch had occurred. Selections of the optimal choice remained significantly lower in block 4 (*M* = 9.31) and block 5 (*M* = 10.36) compared with block 2 (all *p*'s < 0.025). No other differences were statistically significant. Neither the main effect of condition nor the block-by-condition interaction was statistically significant, for both *F* < 1. We, therefore, fail to find support for H_3_ that providing participants with a history of the options' outcomes leads to slower adaption following the switch.

#### Outcome SC

To examine H_4_, we first regressed SC outcome on the outcome obtained (0 = −10, 1 = +10) and found that outcome SC was greater following a negative outcome than a positive outcome, *b* = −0.044, *z* = −2.38, *p* = 0.017. This supports the SMH and H_4_, which predicted that outcome SC would be greater following a negative outcome compared with a positive outcome. To control for the length of time playing the task, and therefore the amount of information obtained about the options, we included the block and its interaction with the outcome obtained in the next step of the regression. The block (variable centered) was now the only significant predictor, with greater outcome SC as the game progressed, *b* = 0.045, *z* = 4.65, *p* < 0.001. Both outcome obtained and its interaction with the block were non-significant, *b* = 0.025, *z* = 0.57, *p* = 0.568, and *b* = −0.017, *z* = −1.25, *p* = 0.210, respectively. Thus, although losses resulted in greater physiological responses, the effect of SC was no longer significant once task experience was controlled for. Therefore, we find some, but inconsistent, support for the predictions of the SMH that losses result in greater physiological reactions than wins.

**Figure 2:**
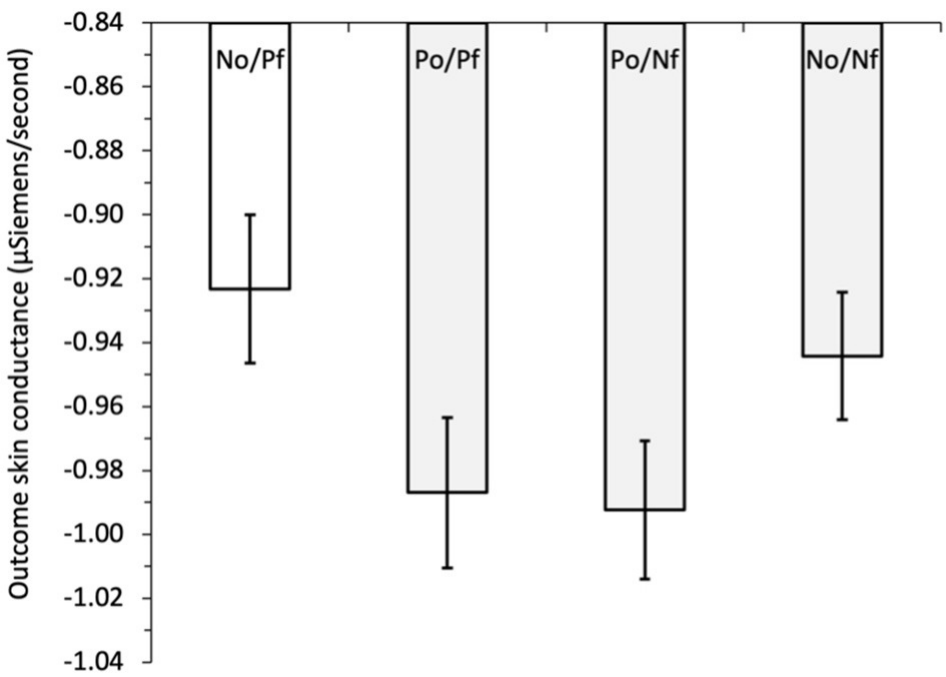
Study 1: mean outcome skin conductance (μSiemens/second) for obtained and forgone outcome combinations: N_O_/P_F_, negative obtained/positive forgone (reference category); P_O_/P_F_, positive obtained/positive forgone; P_O_/N_F_, positive obtained/negative forgone; N_O_/N_F_, negative obtained/negative forgone. The error bars show the standard error of the mean.

To examine hypothesis H_5_, that the forgone outcomes can affect the size of outcome SCs, we initially regressed outcome SC on outcome combination, i.e., the combination of obtained and forgone outcomes, see Figure 2. The outcome combinations were dummy coded with a reference category of negative obtained/positive forgone (N_O_/P_F_), vs. positive obtained/positive forgone (P_O_/P_F_), positive obtained/negative forgone (P_O_/N_F_), and negative obtained/negative forgone (N_O_/N_F_). The outcome SC was lower in all outcome combinations compared with the reference category of negative obtained/positive forgone, indicating greater physiological response when the option selected resulted in a negative outcome *and* the forgone option resulted in a positive outcome. These differences were significant when N_O_/P_F_ was compared with P_O_/P_F_*, b* = −0.073, *z* = −2.71, *p* = 0.007, and to P_O_/N_F_, *b* = −0.067, *z* = −2.54, *p* = 0.011; but not (quite) for comparison with N_O_/N_F_, *b* = −0.048, *z* = −1.84, *p* = 0.066.^4^ Controlling for the amount of information obtained by adding block (variable centered) and its interaction between outcome combination (dummy coded) in the next step resulted in outcome SC being significantly lower for all outcome combinations compared with the reference category of N_O_/P_F_: vs. P_O_/P_F_, *b* = −0.062, *z* = −2.24, *p* = 0.025; vs. P_O_/N_F_, *b* = −0.063, *z* = −2.38, *p* = 0.017; and vs. N_O_/N_F_, *b* = −0.068, *z* = −2.55, *p* = 0.011. The main effect of the block was also significant, *b* = 0.047, *z* = 3.42, *p* = 0.001, indicating a tendency for outcome SC to increase across the blocks of the game. The interactions between the outcome-combination dummy variables and block were all non-significant (all *z* < 1.46, all *p* > 0.145). Thus, consistent with claims that regret is a powerful emotion, observing that a better outcome could have been obtained leads to increased outcome SC compared with all the other combinations of obtained and forgone outcomes.

#### Anticipatory SC

**Figure 3:**
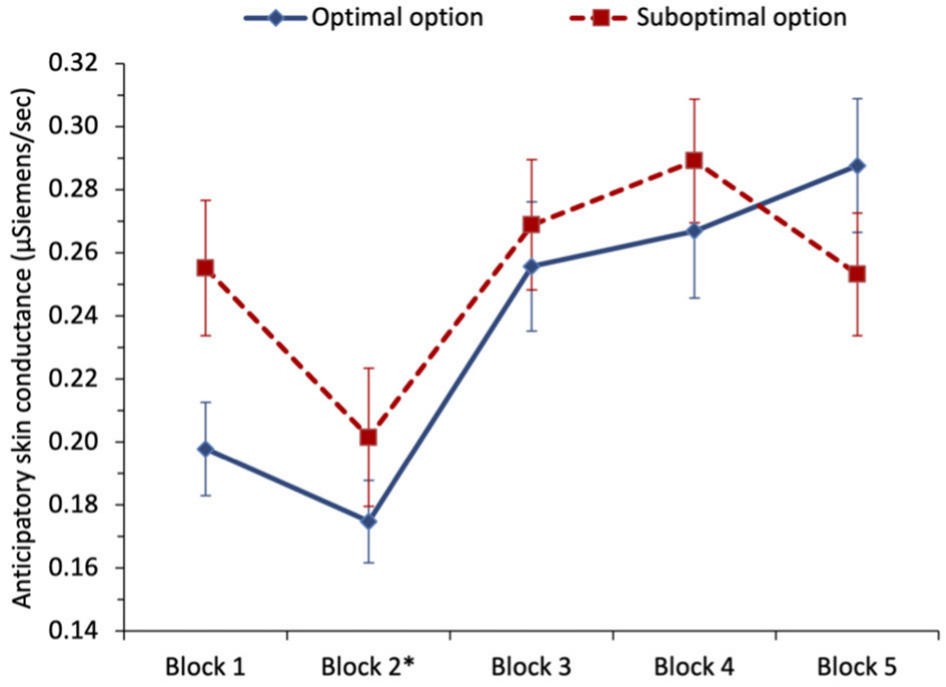
Study 1: mean anticipatory skin conductance (μSiemens/second) for optimal and suboptimal selections by block. The error bars show the standard error of the mean. The asterisk denotes that a switch in payoffs occurred at the end of block 2.

To examine whether anticipatory SC predicted optimal selections in the task (H_6_), we ran two separate multilevel logistic regressions for each of the two different optimal phases of the task: the first phase included blocks 1 and 2 where the non-stationary option was optimal, and the second phase included blocks 3–5 which followed the switch to the stationary option becoming optimal, see Figure 3. For the first phase, we regressed optimal selection (0 = suboptimal, 1 = optimal), on anticipatory SC (variable centered). We found that higher anticipatory SC predicted selecting from the suboptimal option, with odds ratio = 0.60, *z* = −2.38, and *p* = 0.017, in support of the SMH and H_6_. In the next step, including block (variable centered) and its interaction with anticipatory SC (variable centered) resulted in the effect of anticipatory SC shrinking and becoming non-significant, with odds ratio = 1.11, *z* = 0.16, and *p* = 0.871. The effect of the block was significant, indicating that optimal selections increased from block 1 to block 2, odds ratio = 2.09, *z* = 5.18, *p* < 0.001. The interaction between anticipatory SC and block was not significant, with odds ratio = 1.40, *z* = 0.85, and *p* = 0.396. During the first 40 trials of the task, participants learned to select more from the optimal option, and although anticipatory SC was predictive of optimal selections, controlling for task experience eliminated this effect.

In phase 2, we initially regressed optimal selection (0 = suboptimal, 1 = optimal) on anticipatory SC (variable centered), but found no significant effect of anticipatory SC, with odds ratio = 0.93, *z* = −0.52, and *p* = 0.601. Following the payoff switch which made the stationary option optimal, there was no difference in anticipatory SC before selecting from either option. Including block and its interaction with anticipatory SC (variable centered) in the next step resulted in the block being the only significant predictor, with optimal selections increasing from block 3 to block 5, odds ratio = 1.30, *z* = 4.32, *p* < 0.001—indicative of reversal learning after the switch. Anticipatory SC remained non-significant and the interaction with the block was also non-significant, with odds ratio = 1.17, *z* = 0.98, and *p* = 0.327. In the last 60 trials of the task, anticipatory SC did not significantly predict optimal selections.^5^

### Discussion

Study 1 examined whether participants could adapt to changes in the probabilities of payoffs and whether somatic states indexed by SC plausibly played a role in aiding reversal learning of the payoff structure. Initial learning in the task was successful, participants' rate of selection of the (non-stationary) optimal option was significantly above chance after 21–40 trials. The optimal option switched at trial 41. Despite the symmetry in our design whereby expected value differences between options were of equal size before and after this switch, participants did not choose as successfully after 60 trials in this new environment as they had after 40 trials in the original task environment. We found no significant effect of showing participants a running history of previous outcomes which has *sometimes* been found to speed up initial learning but also to increase inertia when the optimal option changes (Rakow and Miler, 2009).

Supporting the SMH, we found significantly greater outcome SC to negative outcomes compared with positive outcomes for the option chosen, though this effect reduced when the time course of the task was taken into account. This limited effect of outcome valence on outcome SC is perhaps surprising given the role that the SMH ascribes to somatic outcome responses in marking options and the subsequent development of successful IGT performance. This could reflect differences between tasks in the relative scale of losses and gains. In the IGT, the largest losses in both disadvantageous decks are either 2.5 or 12.5 times the size of the $100 nominal reward, whereas, in our non-stationary task, the losses and gains were equal. Although the SMH does not predict greater outcome SC to the greater punishments, the SCR data from the IGT reported by Bechara et al. (1996) seem to show that punishment SCR only reliably exceeds reward SCR in decks where the absolute size of rewards exceeds that of punishments (Decks B and D). This is consistent with the possibility that SC marks risk rather than reward (Tomb et al., 2002). Another possibility is that—because our participants observed both obtained and forgone outcomes—obtained losses have a different status in our task because participants see that, sometimes, they would not have done better by selecting the other option. Indeed, consistent with that we found greater outcome SC following a positive forgone outcome when the obtained outcome was negative, compared with all other combinations of positive and negative outcomes, a finding that remained (in fact, strengthened slightly) when controlling for block number.

The SMH's key prediction is that after experiencing different outcomes, anticipatory SC develops and is greater before selecting from the disadvantageous options than advantageous options. This facilitates advantageous decision-making by helping to avoid previously experienced negative outcomes. We found that anticipatory SC was greater before a non-optimal selection (disadvantageous option) by the end of the first 40 trials. This is in the direction the SMH would predict, with a greater physiological response before picking from a disadvantageous option. This effect was no longer significant once the block number was included in the regression. We did not find any support for a difference in anticipatory SC between the optimal and non-optimal options developing after the switch, in the last three blocks of the game with or without controlling for block number. However, we note that, on average, decision-making was not particularly successful in this latter phase that followed the switch.

To further test the role of anticipatory SC to help guide optimal decision-making, we ran a second study. In light of participants' modest adaptation to changes in payoffs, we added more trials post-switch to allow for more opportunity to adapt to change. Additionally, to provide further opportunity to examine reversal learning, we added a second switch-point at which the optimal option changed. To accommodate these design changes, the length of the game was doubled.

## Study 2

Study 2 employs a similar non-stationary payoff game to Study 1 but uses a 200-trial version with two instantaneous switches. The switches occur at trial 41 where the non-stationary option changes from being the optimal to the suboptimal choice and at trial 121 when the non-stationary option changes to be the optimal choice again. The payoff distributions for both the stationary and non-stationary options are the same as in Study 1. Based on Rakow and Miler (2009) and the results from Study 1, we expect that participants will have developed a preference for the optimal (non-stationary) option within the first 40 trials:

**H**_**1**_**:** There will be a preference for the optimal option by the second block of 20 trials.

Based on Rakow and Miler (2009) and Study 1, we expect initial learning to be more successful than reversal learning. Therefore, even with an extra 20 trials after a switch compared with Study 1, we predict fewer selections for the optimal option in the fourth 20-trial block following a switch than in the second block from the start of the game:

**H**_**2a**_**:** The number of selections from the optimal option for trials 101–120 will be less than for trials 21–40.

**H**_**2b**_**:** The number of selections from the optimal option for trials 181–200 will be less than for trials 21–40.

We again employ a between-subjects design providing a running history to half of the participants and testing whether it moderates participants' adaptation to a change in the payoff distributions:

**H**_**3**_**:** Participants provided with a history of the options' outcomes adapt more slowly following the switches, making fewer selections of the optimal option compared with participants without the history.

We again test whether, as per the SMH, participants have greater outcome SC for the negative outcomes in the task. We also take advantage of our task design to test whether forgone outcomes moderate outcome SC, reflecting affective impulses associated with regret:

**H**_**4**_**:** Outcome SC will be greater for negative outcomes (−10) than for positive outcomes (+10).

**H**_**5**_**:** Obtaining a negative outcome when the forgone outcome is positive will result in greater outcome SC compared with other combinations of obtained and forgone outcomes.

In Study 1, we found mixed support for the SMH's key prediction that decision makers develop anticipatory SCRs before picking from the options, which are greater for disadvantageous options than advantageous ones. We again examine whether, consistent with the SMH, anticipatory SC predicts optimal selections in each of the three phases of the task:

**H**_**6**_**:** Anticipatory SC responses develop and are greater for selections of the suboptimal option (whichever option that is for a given phase of the task).

### Method

#### Participants

There were 36 participants (19 women) recruited from the University of Essex, Psychology Department's Volunteer list. Their mean age was 26.28 years (*SD* = 8.55, range 19–62, IQR 21–28).

#### Apparatus

The SC activity was recorded and analyzed as in Study 1 using Mind Media NeXus-10 and Ledalab software (Benedek and Kaernbach, 2010).

#### Materials and design

The computerized “Money Machine Game” from Study 1 was used, but the number of trials was increased to 200. The machines delivered a win of +10 or a loss of −10. One machine (stationary) had a fixed probability of 0.5 of winning for all 200 trials. The other machine (non-stationary) had a probability of 0.7 of winning for the first 40 trials, which switched to a 0.3 probability from trial 41 onwards to trial 120, and from trial 121 returned to 0.7 for the remaining 80 trials. Participants were randomly assigned to either the experience-only or experience-plus-history condition.

#### Procedure

The procedure and instructions were identical to Study 1, except that a (longer) 5-min baseline was recorded for SC and the instructions (see Appendix) were amended to state: “You will have 200 ‘goes' to win points.” The study session lasted ~40 min, for which participants received UK£4 (or course credit).

### Results

**Figure 4:**
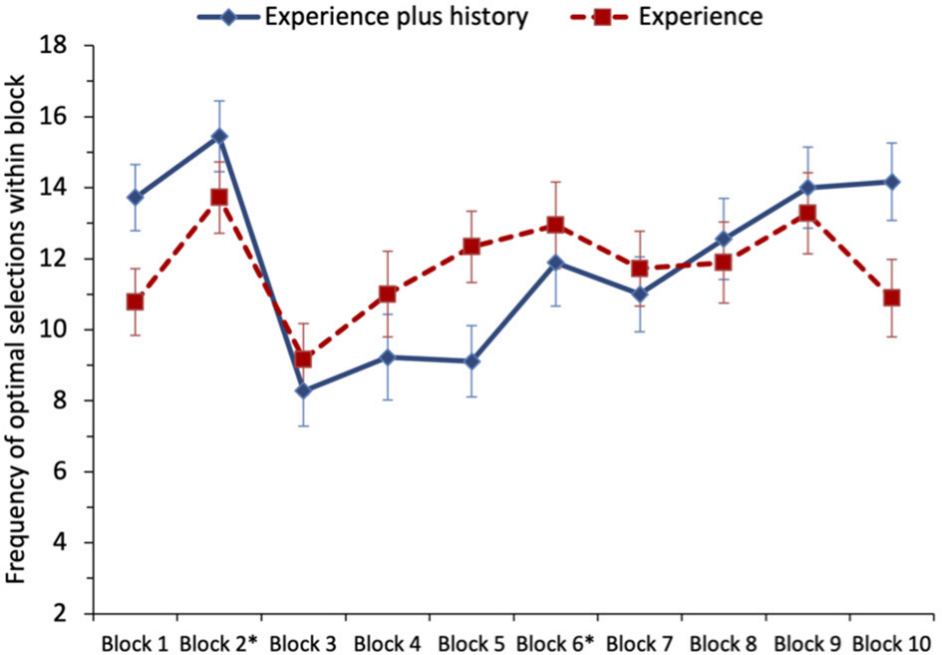
Study 2: mean frequency of selection of the optimal option (non-stationary option in blocks 1 and 2, stationary option in blocks 3–6, and non-stationary option in blocks 7–10) for both conditions of the task (experience only and experience plus running history). Switches occurred at the end of blocks marked with an asterisk. The error bars show the standard error of the mean.

A one-sample *t*-test testing H_1_ found that the mean number of selections from the non-stationary option in block 2 (trials 21–40; *M* = 14.58) was significantly >10 (at-chance performance), *t*_(35)_ = 6.44, *p* < 0.001, *d* = 1.07. This large effect supports H_1_. A paired-sample *t*-test comparing the number of optimal selections (from the stationary option) in block 6 (trials 101–120; *M* = 12.42) to the optimal selections (from the non-stationary option) in block 2 (*M* = 14.58) revealed no significant difference, *t*_(35)_ = 1.90, *p* = 0.066, *d* = 0.32. Thus, while descriptively the means supported H_2a_, there was no statistically significant support for this hypothesis. A paired-samples *t*-test comparing the mean number of optimal selections in block 2 (*M* = 14.58) to those in block 10 (trials 181–200; *M* = 12.53) revealed significantly fewer optimal selections in block 10 compared with block 2, *t*_(35)_ = 2.08, *p* = 0.045, *d* = 0.35. This provides significant support for H_2b_. We note that the means for blocks 6 and 10 were similar and that the differences in significance for the *t*-tests for H_2a_ and H_2b_ represent results falling narrowly on opposite sides of the threshold for statistical significance. Taken together, these data suggest some success in reversal learning, but that preferences for the optimal option following a shift are less clear than those that develop quickly at the start of the task, see Figure 4.

To examine the effect of providing participants with a running history, the number of times the optimal choice was selected was analyzed as 10 blocks of 20 trials. A repeated-measures ANOVA was performed with the number of times the optimal option was picked as the dependent variable and block and condition (experience-plus-history vs. experience-only) entered as factors. The main effect of block was medium-sized and statistically significant, *F*_(3.86, 131.12)_ = 5.17, *p* = 0.001, $\eta_{p}^{2}$ = 0.132. Planned comparisons between the adjacent phases in the game were conducted. Comparing the first 40 trials (blocks 1–2) when the non-stationary option was optimal, vs. the next 80 trials (blocks 3–6) when the stationary option was optimal, revealed significantly fewer optimal selections over the 80 trials following the first switch, *F*_(1, 34)_ = 11.43, MSe = 410.28, *p* = 0.002, *d* = 0.56. This supports H_2a_. Although not testing one of our pre-defined hypotheses, comparing the 80 trials following the first switch (blocks 3–6) vs. the next 80 trials following the second switch (blocks 7–10) revealed no significant difference for the mean number of optimal selections, *F*_(1, 34)_ = 3.50, MSe = 272.22, *p* = 0.070, *d* = 0.31.

There was no main effect of condition, with no difference between the participants who only experienced the outcome and those who also had a history of previous trials, *F* < 1. The block-by-condition interaction was not significant, *F*_(3.86, 131.12)_ = 1.94, *p* = 0.111;^6^ thus, there is no significant support for H_3_ (slower adaption to the switches in the optimal option when provided with a running history). Thus, there is no clear support for the possibility that adding some descriptive data changes task performance in this decision-from-experience task.

#### Outcome SC

To examine H_4_, we first regressed outcome SC on outcome obtained (0 = −10, 1 = +10), and found it was a significant predictor, *b* = 0.028, *z* = 2.23, *p* = 0.025, with greater outcome SC following a positive outcome (*M*_+ve_ = −1.19; *M*_−ve_ = −1.21). This effect was in the opposite direction to that which was predicted, and had been found in Study 1. We then included the block (variable centered) and its interaction with the outcome obtained in the next step. The block was a significant predictor, *b* = −0.0070, *z* = −2.13, *p* = 0.033, while the outcome obtained remained significant and in the same direction, *b* = 0.029, *z* = 2.44, *p* = 0.015 (unlike in Study 1 where the effect was no longer significant). Outcome SC decreased as the blocks progressed; however, the interaction with block and outcome SC was not significant, *b* = 0.00065, *z* = 0.15, *p* = 0.879. We again fail to find support for the predictions of the SMH (and H_4_), which assumes that punishments should result in a greater physiological reaction than rewards.

**Figure 5:**
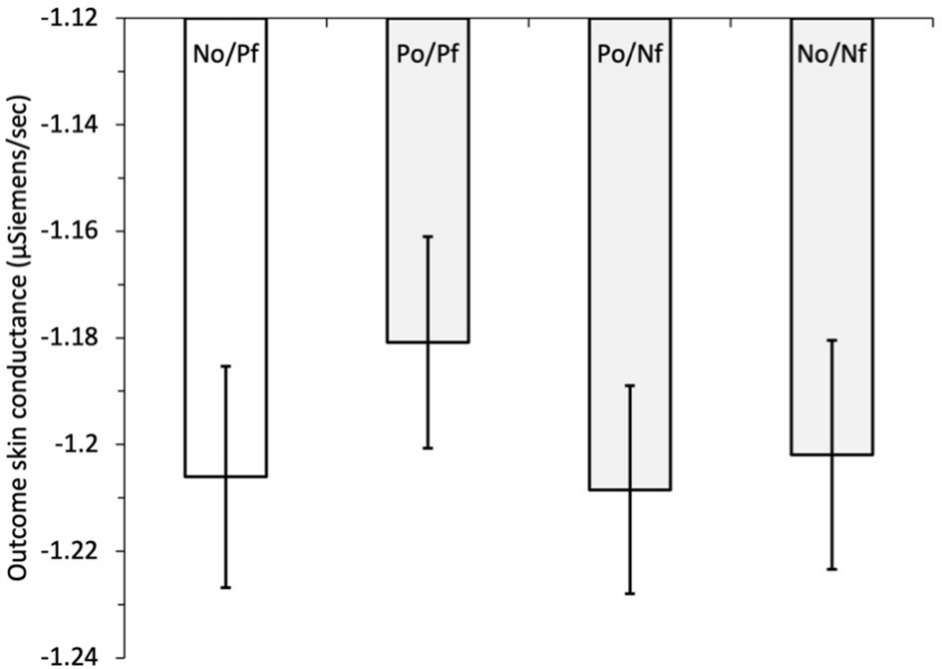
Study 2: mean outcome skin conductance (μSiemens/second) for obtained and forgone outcomes: N_O_/P_F_, negative obtained/positive forgone (reference category); P_O_/P_F_, positive obtained/positive forgone; P_O_/N_F_, positive obtained/negative forgone; N_O_/N_F_, negative obtained/negative forgone. The error bars show the standard error of the mean.

To examine hypothesis H_5_, that the forgone outcome plays a role in SC responses, we initially regressed outcome SC on outcome combination. The possible combinations of obtained and forgone outcomes were dummy coded with a reference category of negative obtained/positive forgone (N_O_/P_F_) vs. positive obtained/positive forgone (P_O_/P_F_), positive obtained/negative forgone (P_O_/N_F_), and negative obtained/negative forgone (N_O_/N_F_), see Figure 5. Outcome SC was significantly *lower* for N_O_/P_F_ compared with P_O_/P_F_, *b* = 0.044, *z* = 2.61, *p* = 0.009, with no significant effects for the comparisons with P_O_/N_F_, *b* = −0.0035, *z* = −0.21, *p* = 0.837, and N_O_/N_F_, *b* = −0.012, *z* = −0.70, *p* = 0.484. This was different to Study 1 in which we found *greater* outcome SC after obtaining a negative payoff while the forgone was positive compared with all the other outcome combinations (P_O_/N_F_ and N_O_/N_F_).^7^ We then added the block (variable centered) and its interaction with the outcome combination in the next step. Outcome SC remained significantly lower when the obtained outcome was negative and the forgone was positive, compared with when the outcome obtained and forgone were both positive. The block was a significant predictor, *b* = −0.0095, *z* = −2.19, *p* = 0.029, indicating that outcome SC decreased across the trial blocks. The interactions between the dummy variable of outcome combination and block were all non-significant (all *z* < 0.47, all *p* > 0.420).

#### Anticipatory SC

**Figure 6:**
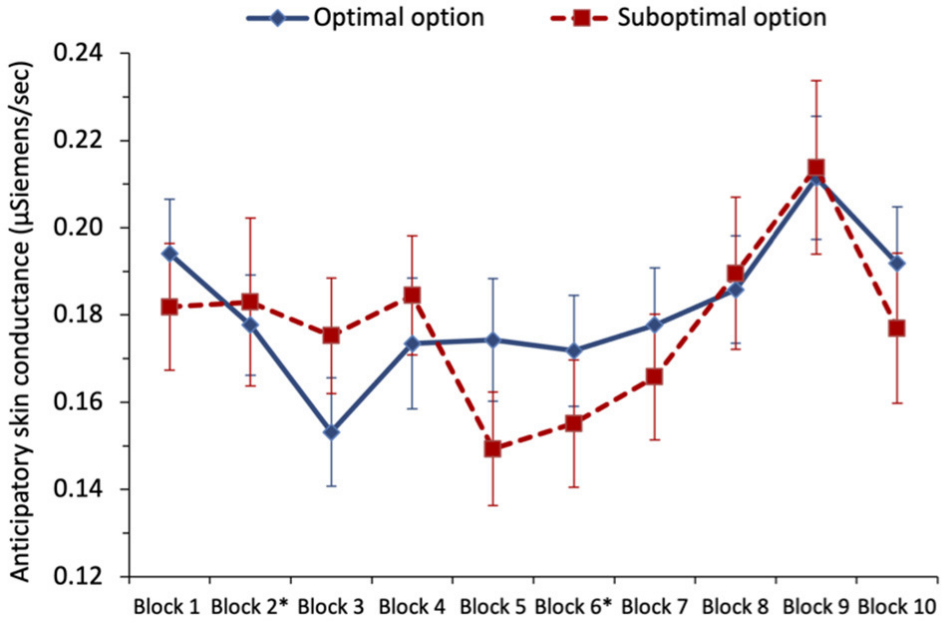
Study 2: mean anticipatory skin conductance (μSiemens/second) for optimal and suboptimal selections by block. Switches occurred at the end of blocks marked with an asterisk. The error bars show the standard error of the mean.

To examine whether anticipatory SC predicted optimal selections, we ran a separate multilevel logistic regression for each of the three phases of the task (i.e., defined according to which option was optimal), see Figure 6. For phase 1, we initially regressed optimal selection^8^ (0 = suboptimal, 1 = optimal) on anticipatory SC (variable centered), but there was no significant effect of SC, with odds ratio = 1.02, *z* = 0.07, and *p* = 0.943. In the next step, we also included block (1–2; variable centered) and its interaction with anticipatory SC. Only block was a significant predictor, with the chances of picking the optimal option increasing from block 1 to block 2, with odds ratio = 1.88, *z* = 5.06, and *p* < 0.001.

In phase 2, regressing optimal selection (0 = suboptimal, 1 = optimal) on anticipatory SC (variable centered), there was again no significant effect of SC, with odds ratio = 0.78, *z* = −1.32, and *p* = 0.188; though this was in the direction predicted by the SMH. Including block (3–6; variable centered) and its interaction with anticipatory SC as predictors in the next step, the block was the only significant predictor, with the chances of picking the optimal option increasing with a block number, with odds ratio = 1.33, *z* = 7.58, *p* < 0.001. The interaction between anticipatory SC and the block was not statistically significant, with odds ratio = 1.30, *z* = 1.74, *p* = 0.081.

In phase 3, when we regressed optimal selection (0 = suboptimal, 1 = optimal) on anticipatory SC (variable centered), there was again no significant effect of anticipatory SC, with odds ratio = 1.10, *z* = 0.56, *p* = 0.578. With the addition of block (7–10; variable centered) and its interaction with anticipatory SC in the next step, again only block significantly predicted optimal choice, with odds ratio = 1.13, *z* = 3.23, and *p* = 0.001. Following the second switch, participants were increasingly likely to pick the optimal option across the blocks, though the effect was descriptively smaller than the equivalent effect in phase 2 (odds ratio 1.13 vs. 1.33 in phase 2).

### Discussion

Study 2 examined whether participants could adapt to two changes in the probabilities of outcomes, and whether physiological signals indexed by SC played a role in aiding learning and reversal learning. Taken together, our analyses reveal a pattern of quick initial adaptation to the task followed by appropriate—though slower—adaptation to changes in payoff structure. Thus, our regression analyses revealed a significant increase in selecting the optimal option across the four trial blocks following a switch in the optimal option. However, at no point did the rate of optimal selection match that of block 2 (just 21–40 trials into the task). We again found no significant effect of providing a running history of the outcomes of both options.

The outcome SC data in this study conflict with the hypotheses we tested and the results obtained in Study 1. In Study 2, outcome SC decreased as the game progressed, rather than increasing as in Study 1. Moreover, when testing H_4_ we found a *greater* outcome SC after a positive outcome compared with a negative outcome. Thus, while SC marked positive and negative events differently, Study 2 did not support the SMH which assumes that punishments result in greater SC than rewards. Examining outcome SC for different combinations of obtained and forgone outcomes, we found that outcome SC was significantly *lower* when the obtained outcome was negative and the forgone was positive, compared with when the outcome obtained and forgone were both positive. This contrasts with Study 1 where we found that outcome SC was significantly *higher* compared with all other possible outcome combinations. In general, outcome SC in Study 2 was greater when the outcome obtained was positive compared with when it was negative, with no clear moderation attributable to forgone outcomes.

We assessed the SMH's key prediction that SC should develop and help guide decision-making through elevated anticipatory SC before selecting a poor option relative to that of a good option. However, examining the three different phases of the game, we never found that anticipatory SC was a significant predictor of picking optimally. Given that our participants evidenced successful decision-making, including reasonably successful reversal learning, anticipatory somatic markers may not be necessary to successfully guide choice in decisions-from-experience. In sum, we found no significant support for the predictions of the SMH in our SC data for Study 2.

## General discussion

In two studies, we used a decisions-from-experience task with a non-stationary option to examine the predictions of the SMH. The switch in which option was superior was analogous to what happens with the disadvantageous IGT deck with low-frequency punishments (“Deck B,” Bechara et al., 1994) where a loss is not obtained until experiencing eight previous high rewards from that deck. This deck has an average net loss but is still often selected (Steingroever et al., 2013). This has led to suggestions that reversal learning is key to optimal performance in the IGT—the ability to “unlearn” that this deck is good. According to the SMH, learning and reversal learning (i.e., “unlearning”) are guided by somatic states that develop with task experience. These somatic states can be indexed by measures of SC. The SMH predicts that SCs measured after an outcome has been obtained should vary dependably with outcome valence, and that SCs measured immediately before a choice should vary with option quality because these anticipatory SCs reflect developing “hunches” about which options are (dis)advantageous. We examined these predictions of the SMH in our SC data, as well as additional predictions about the role of forgone outcomes in somatic responses to outcomes.

### Learning and reversal learning

Participants in both studies, learned quickly which option was superior—their choices revealed a clear preference for the option with a 70% chance of winning over an option with a 50% chance of winning. When the non-stationary option switched to only a 30% chance of winning, participants increased their frequency of selections from the newly superior stationary option. However, their preference for the optimal option was weaker than during the first 40 trials. In general, our results accord with Rakow and Miler (2009; Experiment 1): participants initially picked more from the optimal options, but were slower to adapt, and picked less from the optimal options following the switch. This pattern suggests that outcomes seen in the first few trials of the task remain influential many dozens of trials later. This is somewhat surprising because models that assume decisions-from-experience rely on a small sample of recent observations generally do a good job of explaining these decisions if full feedback is provided as it was in our study (Erev et al., 2010). One possibility is that attention to outcomes reduces with task experience. Supporting this possibility, an eye-tracking study by Ashby and Rakow (2016) found that attention to both obtained and forgone outcomes reduces (lengthy) decisions-from-experience tasks. Additionally, a sizable minority of participants in binary choice tasks adopt a “policy” of selecting the option they judge (or know) to be the best bet on average for all trials from some point in the task (e.g., Newell and Rakow, 2007; Rakow et al., 2010; Newell et al., 2013). Such a “maximizing” strategy is, of course, optimal if payoff probabilities are stationary. Consequently, in such environments, there is no need to continuously monitor or tally outcomes or to update beliefs about the options. Therefore, one explanation for the modest reversal learning seen in our studies is that some participants gave little thought to the possibility that outcome probabilities might change and so gave limited attention to outcomes in the later stages of the game.^9^

Unlike Rakow and Miler (2009), we did not find that providing a running history affected performance. This suggests that experienced information takes precedence over descriptive information in this kind of task. A small number of studies have compared experience-only, description-only, and experience-plus-description conditions. Perhaps the most instructive of these for understanding our data is a series of studies conducted by Weiss-Cohen and colleagues using variants of the IGT, which included conditions that provided descriptions of each deck's payoffs. These descriptions improved task performance (i.e., increased selections from good decks) but only under some conditions. When the IGT was simplified to having two decks with two outcomes per deck, descriptions made no difference to performance (Weiss-Cohen et al., 2018). When descriptions were introduced after participants already had 20, 40, 60, or 80 rounds of experience, the later the descriptive information was introduced the less impact it had (Weiss-Cohen et al., 2021). Our task was similarly simple and only provided descriptive information that exceeded what participants could remember once they had already accumulated some task experience. Therefore, quite possibly, few participants saw any need to use the history that was provided. We conclude that the effects of history that Rakow and Miler (2009) reported are likely not reliable with this kind of task.

### Forgone outcomes and regret

The task we used differs from many other repeated-choice tasks because we also presented the outcome that could have been obtained from the unselected option. In Study 1, the SC response to missing out on a potential positive outcome when the obtained outcome was worse was significantly greater, compared with the obtained outcomes that were either better or equally poor compared with the forgone outcome. This elevated SC could be a physiological representation of regret, similar to loser regret reported in auction studies (Astor et al., 2011). However, we failed to find this in Study 2, where, mirroring the elevated SC to a positive rather than negative outcomes, SC was greater following a positive obtained outcome when the forgone outcome was also positive, compared with all other obtained and forgone outcome combinations. These outcome SC results from Study 2 are not indicative of regret but instead would indicate marking successes with greater emotional arousal (“joy”). It seems that SC responses to positive and negative events are variable.

This variability in the findings across our studies reflects a corresponding variability across studies in the apparent impact of forgone outcomes. Providing forgone outcomes in decisions-from-experience has not always been found to have a strong impact on selections (e.g., as summarized by Yechiam and Busemeyer, 2006). Data from Rakow et al. (2015) clarify that the impact of forgone outcomes of choice is likely context-specific, with forgone outcomes having a greater impact when all options are poor than when all options are good. Put simply, if one is satisfied with the outcome of one's choice (as is likely the case when all options are good), there is less need to consider whether one could have done better by selecting a different option.

### Skin conductance and the SMH

Our study provided an important re-examination of the SMH using skin conductance (SC) data, something which has been surprisingly uncommon (see Dunn et al., 2006; Simonovic et al., 2019) given the importance of SC data in the initial tests of the SMH (e.g., Bechara et al., 1996). Table 1 summarizes our SC hypotheses and results for both studies. The outcome SC following a positive or negative outcome provided mixed support for the SMH. In Study 1, participants had elevated SC to losses, as predicted by the SMH, but controlling for the amount of information acquired in the task (block number) removed this effect. However, in Study 2, gains resulted in elevated SC, in the opposite direction predicted by the SMH and, overall, SC decreased over the course of the game. It is unclear whether outcome SC should persist throughout the task at a similar magnitude or could decrease over time. For example, Bechara et al. (1997) did not report outcome SC but commented that anticipatory SC reduced across the game for the advantageous decks. This mixed pattern of physiological responses to positive and negative outcomes suggests caution is needed when interpreting SC measurement.

**Table 1:** Support for skin conductance (SC) hypotheses (H_4_ to H_6_) for Study 1 and Study 2.

**Hypotheses**	**Study 1 (100 trials)**	**Study 2 (200 trials)**
Outcome SC	Regression	Regression
H_4_: Punishment > Reward	✓	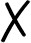
${\text{H}}_{5}^{*}$: Negative obtained (NO) with Positive forgone (PF) > {PO/PF, PO/NF, NO/NF}	✓	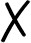
Anticipatory SC	Regression	Regression
H_6_: Phase 1: Suboptimal > Optimal	✓	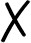
H_6_: Phase 2: Suboptimal > Optimal	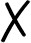	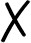
H_6_: Phase 3: Suboptimal > Optimal	N/A	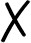

^*^H_5_ is not a hypothesis derived from the SMH.

Greater anticipatory SC has sometimes been reported before selecting good options in the IGT. One example comes from Tomb et al. (2002) who found elevated anticipatory SC before selecting their modified good decks (high reward and high punishment) and suggested anticipatory SC was driven by high rewards, rather than “goodness” or “badness” as suggested by the SMH. However, Damasio et al. (2002) believe that the result from Tomb et al. can still be interpreted within the assumptions of the SMH because somatic markers can also be positive and therefore help in the selection and promotion of approach strategies. Damasio et al. suggest that the higher anticipatory SC before the good decks in Tomb et al.'s modified task may reflect a positive somatic state that promotes the approach. Tomb et al. (2002) did not report the outcome SC, so it is unclear whether the modified good decks also resulted in significantly greater outcome SC. The elevated SC to positive decks in Tomb et al.'s modified version was claimed to be a result of the immediate reward, which was much greater compared with the rewards of the bad decks. The rewards and punishments in our decisions-from-experience task did not change between Study 1 and Study 2, however, we still found greater outcome SC to the positive outcomes (+10) in Study 2, but the reverse (and expected) pattern was found in Study 1, see Table 1. Notably, we find these differences between our two studies despite their very similar design, procedures, and participant recruitment. We will resist the temptation to “explain” the between-study variance in our findings by appealing to what small differences did exist between our two studies because only with formal *within-experiment* manipulation could we be confident about such attributions.

The lack of strong support for the role of anticipatory SC in our decisions-from-experience tasks further questions the generalizability of the SMH. Only in the first 40 trials of Study 1 did anticipatory SC predict optimal choice, in support of the SMH; however, when block number was included in analyses, this effect was no longer significant and we failed to detect differences in anticipatory SC after the switch, or in any phases of Study 2. Although optimal play was lower after a switch occurred, and remained lower compared with the initial 40 trials, participants did show statistically significant adaption to the new optimal option in both studies (as evidenced by the regression analyses). However, our SC measurement could not identify any way in which this reversal learning was accompanied by changes in anticipatory somatic states.

The SMH represents one of several prominent accounts of decision-making that assumes a key role for affect in decision-making (Loewenstein et al., 2001). A key consideration in this literature is the balance between cognitive and affective processes in different kinds of decisions (Slovic et al., 2004; Figner et al., 2009). Reflecting these considerations, and relevant to our data, Glöckner et al. (2012) assessed physiological arousal in both decisions-from-description and decisions-from-experience to examine whether the different situations activate different affective or cognitive processes. Examining arousal by using SC,^10^ they found that the difference in the expected value between the two gambles predicted peak arousal in SC in the description condition but not in the experience condition. In the description condition, as the difference between the expected value of the gambles increased, SC decreased when considering the description of the gambles. Thus, the data from Glöckner et al. (2012) raise the possibility that emotional responses may differentiate options more clearly in decisions-from-description than they do in decisions-from-experience.

Important data from Fernie and Tunney (2013) provide another perspective on the balance between cognitive and affective processes in experience-based choice. Fernie and Tunney collected SC measures for the IGT and asked their participants to report on their task knowledge every 10 trials. Participants performed well at the task. However, counter to the SMH, Fernie and Tunney found no evidence that anticipatory SC differentiated between the decks. This was true at all stages of the task, both before and after the point where participants could articulate knowledge of deck quality. Although Fernie and Tunney reported that participants' conceptual knowledge of the task was weak, most participants demonstrated partial task knowledge. Typically, this began to emerge around the 20th trial. In sum, these data did not support the claim that somatic markers are important for choosing well in the IGT. But also, an advanced state of knowledge was not required for good task performance.

### Limitations, alternative interpretations, and future directions

Our investigation used skin conductance measures collected from a choice task with non-stationary payoffs to test the SMH. Across several such tests, our data failed to corroborate the SMH. One might conclude from this that the SMH is wrong. Relevant to such an interpretation, however, Chalmers (1999, p. 89) summarizes the conundrum faced by the scientist who has collected observations that contradict the predictions of a theory:

“It may be that the theory under test is at fault, but alternatively it may be that an auxiliary assumption or some part of the description of the initial conditions that is responsible for the incorrect prediction. A theory cannot be conclusively falsified, because the possibility cannot be ruled out that some part of the complex test situation, other than the theory under test, is responsible for an erroneous prediction.”

This problem (known as the Duhem–Quine thesis) points to alternative interpretations of our data, several of which are linked to our choice of methods or study limitations.

First, our failure to corroborate the SMH may be attributable to our choice of research task. In contrast to the IGT, we provided forgone feedback from the non-chosen outcome. Such tasks afford learning processes that circumvent the need to evaluate options independently. For example, one can do well at the task by switching options after a relative loss (i.e., obtained outcome < forgone outcome) but otherwise sticking with the same option (Kareev et al., 2014). If the task were approached in this way, mental representations of individual option quality are unnecessary. This would make somatic markers redundant for learning. Therefore, one possibility is that the SMH describes processes that aid learning in tasks that require independent evaluations of different options, but which are irrelevant to some other types of choice task (such as ours). Future research should test this conjecture by manipulating task structure or task conditions experimentally (e.g., manipulating the presence of forgone feedback).

Second, it may be that the “failure” of the SMH in our studies was due to how we measured somatic responses. Skin conductance is a well-established index of affective response for decision research (Figner and Murphy, 2011). However, as discussed above, there are difficulties identifying exactly what aspect(s) of affect SC reflects (e.g., Tomb et al., 2002). These difficulties may be compounded by the fact that there are different ways to extract and analyze SC responses, which we cannot guarantee will always result in the same conclusion. Moreover, even though we used recommended procedures consistently in both studies, our SC data varied between studies in ways that we cannot easily account for. Therefore, to clarify the role of somatic markers and affective processes in risky choice, it would be wise to employ a range of measures (e.g., heart rate and pupil dilation) that afford additional tests of the SMH.

A further problem with our measurement was the limited statistical power of our investigation. Our sample sizes (*N* = 36 per study) were chosen to be similar to those in some of the SC studies that informed our investigation and were constrained by our available resources of time and money. With the benefit of what we have since learned, we now appreciate that our studies had far less statistical power than was desirable. Moreover, it seems this is true of most (perhaps all) studies that have used SC to test the SMH. The meta-analysis by Simonovic et al. (2019) examined two effects that the SMH predicts for the IGT, which are measurable by anticipatory SC. They report a mean effect of *r* = 0.22 (*k* = 15, total *N* = 1,147) for the relationship between anticipatory SCR magnitude and IGT performance, and a mean effect of *r* = 0.10 (*k* = 8, total *N* = 678) for the difference in anticipatory SCR between good and bad decks. It is this latter small effect that we examined in our anticipatory SC data (hypothesis H_6_). To achieve 80% power with α = 0.05 (two-tailed), a sample of *N* = 783 is required to detect an effect of *r* = 0.1 (Cohen, 1992). This rises to *N* = 1289 for 95% power (e.g., as required by *Nature Human Behavior* for Registered Reports). Even for *r* = 0.2, achieving 80 or 95% power requires sample sizes of *N* = 189 or *N* = 314, respectively. Moreover, testing the boundary conditions of a theory often requires testing *differences* between effects (Nieuwenhuis et al., 2011). An example of this would be when testing whether the relationship between anticipatory SC and option selection differs between tasks with and without forgone outcome feedback. For such designs, *N* = 1,573 is required for 80% power to detect a difference of 0.1 between two correlations. Given this state of affairs, the meta-analysis by Simonovic et al. (2019) suggests that only one published IGT study in a non-clinical population can be said to *approach* adequate statistical power to test the SMH via SC data. Specifically, Ottaviani and Vandone (2015) tested *N* = 445 participants, and no other study in the meta-analysis tested more than 135 participants.

In sum, despite 30 years of research into the SMH, it appears that further research is required to provide adequate direct tests of its key claims about how somatic markers guide decisions. Such tests should include multiple measures of affective response and anticipatory affect, including—but not restricted to—skin conductance measures. Some of these studies should also include different tasks (within the same study) and manipulate the conditions for the study task(s) so that any boundary conditions of the SMH can be mapped out. To meet current expectations for reproducible behavioral science, we recommend a *minimum* sample size of 750 *per condition*. For example, an investigation of two tasks, each examined via two measures of affect, would require *N* > 3,000 if the measures cannot be collected concurrently and the tasks are compared between-subjects. Such studies are likely beyond the resources of any one research lab and only achievable through collective research efforts, such as those afforded by collaborative networks such as the *Psychological Science Accelerator* (Beshears et al., 2022) or the *Reproducibility Project in Psychology* (Chan et al., 2022).

## Summary

In two studies, we examined decisions-from-experience in a two-option task with non-stationary payoffs. The non-stationary nature of the task mirrors the set-up of the initially appealing, disadvantageous Deck B in the IGT, for which the SMH proposes that somatic states develop after experiencing severe losses in some of the later card selections. The SMH further proposes that somatic markers help guide players away from selecting it—to avoid repeating previous mistakes. Our SC measurements showed little support for these hypothesized roles of somatic states in the successful initial learning and moderately successful reversal learning displayed by our participants. We conclude that this type of task is suitable for testing the SMH, while acknowledging that there is scope for better tests of the SMH in future studies.

## Data availability statement

The datasets for the studies reported in this paper are available at https://osf.io/qe2w7/.

## Ethics statement

The studies involving human participants were reviewed and approved by Faculty Ethics Committee (Faculty of Science) University of Essex. The participants provided their written informed consent to participate in this study.

## Author contributions

RW and TR collaborated on the conception and design of the studies, the interpretation of the findings, and the writing of this manuscript. RW programmed the study tasks, carried out the studies, and processed and analyzed the data. All authors contributed to the article and approved the submitted version.

## Footnotes

^1^A preference for the disadvantageous Deck B in the IGT represents an extreme example of such an underweighting of rare losses. This preference has been observed among a sizeable proportion of participants in several IGT studies (for a review, see Steingroever et al., 2013).

^2^Based on university entry requirements, we expect participants' educational attainment and years of education to be above the average for the general population. However, from measures taken in other studies, we know that this population of volunteers from which our sample came varies widely on specific cognitive abilities (e.g., abstract reasoning and numeracy) that could be relevant to this task. For example, in a study with 160 participants from this research volunteer pool, (Heard et al., 2018) assessed numeracy via the Berlin Numeracy Test which places individuals into one of four quartiles determined by general population norms (Cokely et al., 2012). Some participants were in each quartile, though around 72% were in one of the *lower* two quartiles.

^3^We are grateful to a reviewer for pointing out that sample sizes in SC studies have increased in recent years. The meta-analysis by Simonovic et al. (2019) provides some descriptive support for this evaluation. Across the 20 studies in the meta-analysis, the rank correlation between sample size and publication year was *r*_s_ = 0.16.

^4^To check for any other differences, all outcomes obtained were entered as the reference category, but no other significant differences were found.

^5^The same pattern of results was found running the analyses in phases but using option chosen as the dependent variable.

^6^The interaction between condition and block was significant when not using Greenhouse–Geisser corrected degrees of freedom, *F*_(9, 306)_ = 1.94, *p* = 0.047.

^7^Changing the reference category to be positive obtained/positive forgone, outcome SC was significantly lower in all other outcome comparisons to the reference category, *p*'s < 0.009.

^8^As in Study 1, the same pattern of results was obtained for these analyses when using option chosen (non-stationary versus stationary) instead of coding for optimal option.

^9^Our participants' modest adaptation to change may surprise readers familiar with the reversal learning tasks used by neuroscientists to examine cognitive flexibility (e.g., D'Cruz et al., 2011). However, in our task and the IGT, suboptimal options can still deliver a win, and optimal options can still deliver a loss, on an individual trial. In contrast, reversal learning tasks in neuroscience are deterministic in that one option (which can switch) always delivers a win. Therefore, our task and the IGT are more complex than the reversal-learning tasks employed in neuroscience, therefore examining a different type of learning.

^10^SC was assessed as the maximum peak arousal within the entire sampling phase, rather than on an individual trial-by-trial basis as reported here.

## Appendix

### Verbatim participant instructions for study 1

You will play a game. In this game you will see two “money machines” like those shown below. When you click on a machine you will win or lose points. Your target is to obtain as many points as possible. You will have 100 “goes” to win points. On each go, you should decide which machine you want to get points from. On each go, the probability of winning or losing points is fixed in advance. However, this probability may change from one go to the next. When you click on a machine, you will see how many points you obtained. You will also see how many points you would have obtained from the other machine. Pay close attention to this information so that you can decide which machine is the best one to play. You will also see your new points total after every go. If you have any questions, ask the experimenter. Press “Ready to begin” to start the first game.
